# Invasive fungal infection by *Aspergillus flavus* in immunocompetent hosts: A case series and literature review

**DOI:** 10.1016/j.mmcr.2018.10.006

**Published:** 2018-10-25

**Authors:** Ana María Garcia-Giraldo, Barbara Lucia Mora, Jorge Mario Loaiza-Castaño, Jorge Andrés Cedano, Fernando Rosso

**Affiliations:** a Facultad de Ciencias de la Salud, Universidad ICESI, Calle 18 # 122-135, Cali, Colombia; b Clinical Research Center, Fundación Valle del Lili, Carrera 98 #18–49, Cali, Colombia; c Facultad de Medicina, Universidad CES, Calle 10A #22–04, Medellín, Colombia; d Infectious Diseases Service, Fundación Valle del Lili, Carrera 98 #18–49, Cali, Colombia

**Keywords:** Aspergillosis, Invasive Aspergillosis, Immunocompetent host, Paranasal sinus

## Abstract

Invasive aspergillosis usually affects immunocompromised hosts with variable manifestations depending on the site of infection. In this article, we present two cases of invasive Aspergillosis in two non-immunocompromised patients; both cases had a paranasal sinuses infection, with intraorbital and intracranial extension, requiring surgery and antifungal treatment with Voriconazole. These cases were initially diagnosed as paranasal sinus neoplasms. However, the pathology and microbiology studies revealed invasive fungal infection by *Aspergillus flavus*

## 1 Introduction

Paranasal sinus aspergillosis (PNSA) can be invasive or non-invasive. The latter is more common, has a better prognosis and can be presented as an allergic sinusitis. This type of infection destroys the mucosa but does not invade the bone tissues [1], [2]. On the other hand, invasive aspergillosis more commonly affects the lung, followed by sinuses and gastrointestinal tract. Additionally, it has a worse prognosis with high morbidity and mortality due to an intracranial and intraorbital extension, with a mortality rate that ranges between 35% and 90%, primarily in the immunocompromised hosts [1], [3].

The invasive form of the infection occurs most frequently in immunocompromised patients such as those with HIV, diabetes mellitus (DM), cirrhosis, cancer, use of immunosuppressive agents or transplantation [4]. There are few reports of invasive aspergillosis affecting immunocompetent patients, features shared by both cases presented below.

As for the diagnosis of this entity, and due to the wide variety of differential diagnosis, multiple studies are required, such as diagnostic images, biopsy for pathological study and microbiological isolation of the fungus. Nevertheless, isolation may take a considerable amount of time and delay the diagnosis [4].

The management of these patients should be multidisciplinary to establish an adequate antifungal treatment with surgical debridement to prevent recurrence and search for factors that are predisposing the infection of this microorganism.

## 2 Case

### 2.1 Case #1

**Fig. 1:**
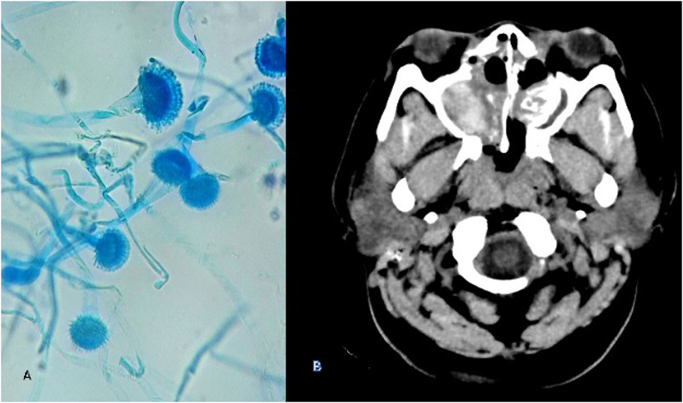
A. KOH with *Aspergillus flavus* septated hyaline hyphae. B. Brain MRI with a nasopharynx-dependent mass invasion of the anterior cerebral fossa, orbit and maxillary and frontal sinuses.

A 42-year-old female patient with past medical history of obesity and a bariatric surgery consults for a year of symptoms consisting in progressive bilateral exophthalmos, especially on the left side, associated with eye pain, bilateral hyaline rhinorrhea and headache, without a change in visual capacity. Upon consultation to the emergency room (day 0), a brain MRI was performed, with a nasopharynx-dependent mass invasion of the anterior cerebral fossa, orbit and maxillary and frontal sinuses finding (see Fig. 1**-B**). At first, it was diagnosed as an invasive nasopharyngeal tumor associated with cerebrospinal fluid (CSF) fistula, which was scheduled for surgical resection.

She was admitted for tumor resection through bifrontal craniotomy with transnasal endoscopic approach (day +69). On admission, the patient was stable, without motor or sensory deficits at the neurological physical exam. After tumor resection, the microbiological study of the lesion was performed, where KOH showed septated hyaline hyphae on day +71 and the culture was positive for *Aspergillus flavus* (day +75) (see Fig. 1**-A**). Pathological analysis reported invasion of all the nasal respiratory mucosa by a granulomatous inflammatory infiltrate, with a few foci of necrosis and extensive areas of fibrosis, giant cells and histiocytes had septated hyaline hyphae phagocytized. The bone fragments were surrounded by the same type of inflammatory infiltrate. The coloring of PAS and Gomori allowed identification of the typical hyphae of Aspergillus in the respiratory mucosa and bone tissue. The patient received amphotericin B 50 mg IV q 24 h from day 70 to day 75, and subsequently, after the identification of A. flavus, management was changed to voriconazole 200 mg PO q12hours during 6 months.

On day +74, multiple extension studies were performed, including a negative serum galactomannan (0.326), a negative HIV serology, normal immunoglobulin G 14.4 g/l, CD4: 660 cells/µl (normal), CD3:1042 cells/µl (normal), and a computed tomography scan (CT scan) of the thorax, where a right apical pulmonary nodule with frosted glass pattern was reported, suggestive of initial infection by Aspergillus. Afterward, bronchoscopy and bronchoalveolar lavage (BAL) were performed (day +76), with galactomannan in BAL reported positive (result: 0.718, positive: >0.5).

Laboratory tests, including complete blood count (CBC), kidney and liver function tests (LFT) were within normal limits during the hospitalization. Protein electrophoresis showed moderate decline in albumin.

During the post-operatory, the patient persisted with a cerebrospinal fluid fistula and received acetazolamide from day +78 to day +98. The remaining clinical course was uneventful, and patient was discharged on day +89 with full recovery and without recurrence.

### 2.2 Case #2

A 76-year-old female patient with a history of hypothyroidism managed with levothyroxine 50 g/day and recurrent left eye chronic dacryocystitis by Methicillin-resistant S. aureus (MRSA) (day −27). Required reconstructive surgery of the left lacrimal pathway (day 0), where a lacrimal duct lesion extending to the adenoid region was found, and resection of the lacrimal duct bone was also performed. The pathology report showed lymphoplasmacytic severe chronic inflammatory infiltrate in soft tissue and fragments of bone with osteomyelitis, as well as a mass that revealed septate hyphae and some irregular, partially pigmented with occasional formation of conidial heads of Aspergillus in the microbiological examination (day +13). In the fungal culture, *Aspergillus flavus* was isolated (day +26); thus craniofacial invasive aspergillosis was diagnosed, and management with voriconazole 200 mg PO q12h was initiated on day +13 and continued for three months (day +103). Extension studies were performed on day +14, which reported a normal CBC, kidney function, and LFT, negative serum galactomannan (0.315), negative HIV, a brain MRI showing microangiopathic leukoencephalopathy without others abnormalities and a thorax CT scan that reported nonspecific thickening of interlobular septae. The patient's clinical course was adequate without recurrence of the disease.

## 3 Discussion

Invasive PNSA can be presented as a local or fulminant form and it is more commonly associated with immunocompromised hosts [2]. Significant risk factors include neutrophil defects, steroid treatment, chemotherapy, AIDS, chronic granulomatous disease and bone marrow or solid organ transplantation [1], [2], [4], which were not present in any of the two cases we presented. The most frequently found Aspergillus subspecies on invasive infections is the *A. fumigatus* species complex [4]. In a study that included 218 cases of infections on transplantation centers in North America [5], 67% of infections were due to members of the *A. fumigatus* species complex, followed by *A. flavus* (13%), *A. niger* (9%), and *A. terreus* (7%). Nevertheless, it is more common to find *A. flavus* in paranasal sinuses and skin infections [4]. Even if the patients we presented were not immunosuppressed, it is possible that specific factors were involved in acquiring the infection. For instance, in the first case, the patient had a history of bariatric surgery, so it is possible that specific nutritional deficits, such as vitamin B12, iron, and folic acid deficit could disrupt the immune system. In the second case, the patient had a previous dacryocystitis infection by Staphylococcus aureus, which according to the previously published literature, could increase the risk of infection by Aspergillus due to a mechanical obstruction of the lacrimal ducts [6].

### 3.1 Differential diagnosis of fungal infections in the immunocompetent host

It is a medical challenge to differentiate between neoplastic disease and invasive fungal infection since they are clinically similar and the imaging findings are not conclusive, hereby biopsies with pathological study are useful in these situations. In the first case, the initial diagnostic impression was a neoplasm, and then pathologic and microbiologic study revealed invasive aspergillosis. Proving that imaging, pathology, and microbiology studies are essential to guide an adequate treatment [7].

Other authors suggest the measurement of galactomannan and β-(1, 3)-d-glucan (BDG), which are components of the fungal cell wall, can be useful in the diagnosis. Galactomannan can be measured in serum or in a bronchoalveolar lavage, and it is relatively specific for Aspergillus, with a 71% sensibility and an 89% specificity [8]. The 2016 IDSA guidelines [2] recommend using it in immunosuppressed patients, excluding its use in chronic granulomatous diseases and solid organ recipients. On the other hand, BDG can be detected in other fungal infections and has a 64% sensibility and 84% specificity [8]. The IDSA guidelines recommend using BDG for invasive aspergillosis in high-risk patients, such as those with hematologic malignancies and allogeneic stem cell transplantation. More recently, the IDSA recommends employment of molecular methods when isolates report atypical growth or concerns for resistance are present [2].

### 3.2 Delayed diagnosis and treatment of invasive fungal infection in the immunocompetent host

A late diagnosis of this condition is related to therapeutic failure and progression of the disease, ultimately leading to complications and death of the patient [9]. In both of the cases reported in this article, there was a delay in the diagnosis. Fortunately, both patients evolved adequately. This delay has also been reported in previous reports, such LaBarbera et al. and Baeesa et al., where appropriate treatment was not started early due to the difficulty in culturing Aspergillus and the unspecific clinical manifestations [10], [11]

In these patients, treatment with voriconazole was initiated, since this is the drug of choice for invasive Aspergillus infections, due to its tolerance, greater effectiveness, and lower toxicity compared to amphotericin B [1], [2], [3], it also has excellent penetration into tissues including central nervous system [12]. Our two cases were treated with this medication plus surgical resection with excellent results.

### 3.3 Other cases reported in the literature and their outcomes

Cases of invasive aspergillosis on immunocompetent hosts have been published previously, Table 1 summarizes 14 published articles so far with the report of 19 cases. The ages of the patients described ranged from 14 to 70 years of age, with 10 female and 9 male patients. The majority of the papers report the isolation of *A. fumigatus* species complex (6 out of 7 cases that reported a species) with a demise outcome in 9 of the total 19 cases included in this literature review. The most common site of infection is the lung and thorax (8/19), followed by head and neck (11/19), especially, paranasal sinuses with further dissemination to the orbit and brain. This finding is consistent with the fact that Aspergillus infects via inhalation of its spores. Therefore lungs, sinuses, and other airway sites may be the most commonly affected sites [13].

**Table 1:** Previous case reports of invasive aspergillosis on immunocompetent hosts.

**Author**	**Age**	**Gender**	**Comorbidities**	**Site of infection**	**Aspergillus species**	**Management**	**Outcome**
**Carpio CJ et al.**[14]	69	Male	Smoker, COPD, Bronchiectasis, Malnutrition	Lung	A. terreus	Voriconazole	Recovery
**Bunkar ML et al.**[15]	19	Male	None	Lung and thorax	A. fumigatus	Voriconazole	Recovery
**Rasoolinejad M et al.**[13]	29	Female	A short period of steroids	Cerebral	Not specified	Amphotericin B then Itraconazole (it recurred, so Voriconazole was initiated)	Recovery
**Moreno-González G et al.**[16]	56	Female	Long Immobilization	Disseminated (lungs, heart, brain, and kidneys)	A. flavus and fumigatus	Voriconazole, caspofungin and amphotericin	Demise
**Abir B et al.**[17]	70	Female	None	Paranasal sinuses	Not specified	Voriconazole	Recovery
**Dimitrakopoulos I et al.**[9]	64	Male	None	Paranasal sinuses, orbit and cranial fossa	Not specified	Amphotericin B and surgical intervention	Demise
**Hersh CM et al.**[18]	55	Female	Alcohol Use	The optic nerve, cavernous sinus, and maxillary sinus	Not specified	No specified	Demise
**Kim JH et al.**[19]	29	Male	None	Lung	A. fumigatus	Voriconazole	Recovery
**Ratermann KL et al.**[20]	51	Female	Near Drowning experience	Lung	A. fumigatus	Amphotericin B and Voriconazole	Demise
**Subramanian S et al.**[21]	40	Male	None	Orbit, brain and pterygopalatine fossa	Not specified	Amphotericin and Surgery	No Follow up
**Kwon OK et al.**[22]	60	Male	Influenza A, HTN	Lung	Not specified	Voriconazole	Recovery
**Bajaj N et al.**[7]	22	Male	None	Heart	A. fumigatus	Voriconazole	Recovery
**Mohammed AP et al.**[1]	33	Female	None	Lungs	A. fumigatus	Caspofungin and Voriconazole	Recovery
**Baeesa SS et al.**^a^[11]	14–53	4 - Female	Not specified	Orbital apex with an intracranial aneurysm and SAH	Not specified	Amphotericin B and Surgery	5/6 demised
		2 - Male					

SAH: Subarachnoid hemorrhage.

a Six patients were included in this study.

In conclusion, the invasive PNSA is an aggressive and uncommon condition in immunocompetent patients, has a high rate of morbidity and mortality even with adequate medical/surgical treatment [9] and requires performing of imaging and pathologic study to obtain a definitive diagnosis given the wide range of differential diagnosis. Moreover, it is essential to perform further studies that elicit the type of immune response immunocompetent patients develop against Aspergillus infection, to understand the mechanisms leading to the disease.
